# Clinical evaluation of toric intraocular lens implantation based on iTrace wavefront keratometric astigmatism

**DOI:** 10.1186/s12886-020-01726-0

**Published:** 2020-11-16

**Authors:** Zhe Zhang, Hui Li, Jing Zhou, Yaqin Zhang, Suhua Zhang

**Affiliations:** Shanxi Eye Hospital, No. 100 Fudong Street, Taiyuan, Shanxi 030001 People’s Republic of China

**Keywords:** Toric intraocular lens, Astigmatism correction, iTrace wavefront aberrometry, Vector analysis

## Abstract

**Background:**

Currently, there is no standard technique for determining corneal astigmatism. The iTrace wavefront aberrometry of cornea calculated steep power and axis based on the best Zernike mathematical fit from all topo data within 4 mm circle. It was supposed to be more accurate than iTrace simulated keratometry which was calculated based on only 4 points on the circle of 3 mm. This aim of this study was to evaluate visual outcomes and rotational stability after toric intraocular lens (IOL) implantation using the wavefront aberrometry of the cornea with iTrace.

**Setting:** Single site in China, Shanxi Eye Hospital, Shanxi, China.

**Design:** Prospective case series.

**Methods:**

The study included 85 eyes of 63 patients undergoing phacoemulsification and toric IOL implantation. The IOL power and cylinders were chosen with the help of the iTrace toric planning program using wavefront keratometric astigmatism. Astigmatic changes were assessed using Alpins vector method over a 3-month follow-up period.

**Results:**

Preoperative mean corneal topographic astigmatism was 1.91 diopters (D) ± 0.69 (standard deviation). Postoperative mean refractive astigmatism decreased significantly to 0.48 D ± 0.34. Surgical induced astigmatism was 1.73 D ± 0.77 and the mean correction index was 0.89 ± 0.22, showing a slight undercorrection. The proportion of astigmatism ≤0.50 D increased from 0 to 71.8% postoperatively.

**Conclusions:**

This is the first study on evaluation of clinical outcomes of toric IOL implantation in corneal astigmatism patients using iTrace wavefront keratometric readings. The findings show that use of iTrace built-in toric calculator is safe and effective for planning toric IOL surgery for wavefront keratometric astigmatism.

**Trial registration:**

Current Controlled Trials ISRCTN94956424, Retrospectively registered (Date of registration: 05 February 2020).

## Background

An estimated 40–50% of the population aged over 60 years has more than 1.0 diopter (D) of keratometric astigmatism [1–3]. Also, 21.3–22.4% of patients with cataracts have 1.0–1.5 D of corneal astigmatism with 10.6–12.4% of patients having 1.5–2.0 D and 8.2–13.0% of patients having 2.0 D or more [4, 5]. Corneal astigmatism management has become crucial in modern cataract and refractive surgery practices. Significant postoperative astigmatism might affect both vision quality and spectacle independence, leading to unsatisfactory outcomes. Toric intraocular lenses (IOLs) have become an increasingly common technique due to their advantage of predictably, stably, and safely correcting a preexisting astigmatism.

Keratometers, corneal topographers, anterior segment tomographers, and intraoperative aberrometers can each provide corneal measurements necessary to accurately predict the ideal IOL cylinder power and alignment meridian to correct astigmatism during cataract surgery [6]. Since each device has its own characteristics, measurements obtained from different devices may not be comparable due to different refractive indices or measurement areas being used. Thus, there is no standard technique for measuring corneal astigmatism.

A wavefront analysis using an iTrace Surgical Workstation (Tracey Technologies Corp., Houston, TX, USA) integrates an aberrometer, corneal topography, and a toric IOL calculator. The iTrace toric IOL calculator offers a choice to match the keratometric values measured by wavefront aberrometry of the cornea or simulated keratometry. The iTrace wavefront aberrometry of cornea calculates steep power and axis based on the best Zernike mathematical fit from all topo data within 4 mm circle. It is supposed to be more accurate than iTrace simulated keratometry which is calculated based on only 4 points on the circle of 3 mm. However, the outcomes of using iTrace toric calculator based on wavefront keratometric (WFK) astigmatism for toric IOL planning must be evaluated. To the best of our knowledge, the present study is the first to investigate the outcomes of toric IOL planning with iTrace toric calculator based on wavefront keratometric astigmatism.

## Methods

### Patients

Institutional review board approval was obtained for the project and this study followed the tenets of the Declaration of Helsinki. After a detailed explanation, informed consent was obtained from each patient prior to enrollment. Prospectively, 85 consecutive eyes of 63 patients having 2.2-mm coaxial microincision phacoemulsification with monofocal toric IOL (AcrySof Toric) implanted were enrolled between May 2018 and February 2019 at the Shanxi Eye Hospital (Taiyuan, Shanxi, China).

Inclusion criteria were cataract patients with preexisting regular corneal astigmatism and wanted a toric IOL implantation; their cylindric values were between 0.75 D and 5.0 D. Exclusion criteria were pregnancy, lactation, irregular corneal astigmatism, diabetic retinopathy, iris neovascularization, congenital eye abnormalities, severe unstable tear film, retinal detachment, glaucoma, pseudoexfoliation syndrome, uveitis, long-term anti-inflammatory treatment, amblyopia, advanced age-related macular degeneration, previous ocular surgery, severe corneal and retinal disease, history of eye trauma and serious intraoperative complications.

### Preoperative assessment

All patients had a full ophthalmologic examination including subjective refraction, uncorrected distance and best-corrected visual acuity measurements, a slit-lamp examination, Goldmann applanation tonometry, and fundoscopy in mydriasis. Ocular biometry was performed using a partial coherence interferometry device (IOL Master 500, Carl Zeiss Meditec AG). Corneal topography was measured using the Oculus Pentacam (Optikgeräte GmbH, Wetzlar, Germany) and iTrace Surgical Workstation. All measurements were acquired in automatic release mode for each eye before using any eye drops or performing other contact-based examinations. Eye alignment evaluations and measurements with good quality (graded as “ok”) obtained via Pentacam, were used in the final analysis. The participant was placed in front of the iTrace and his or her head was carefully aligned with the chin and forehead fixed with the help of an assistant. All measurements were performed in a semidark room with undilated pupils. A single experienced operator (JZ) performed all examinations.

The aberrometer iTrace was used for the wavefront analysis. It uses the ray-tracing principle, sequentially projecting 256 near-infrared laser beams into the eye in a specific scanning pattern; parameter detection takes less than 200 ms. Topographies were captured using the Placido based corneal topographer mounted on the same device. Corneal aberrations were calculated using anterior topography data; internal aberrations were calculated by subtracting the corneal wavefront aberrations from those of the entire eye measured by the ray-tracing aberrometer using the built-in program [7].

### Intraocular lenses

AcrySof Toric IOL (Alcon Labs, Fort Worth, TX) is a one-piece hydrophobic acrylic lens. The optic is measured to be 6.0 mm and can be inserted through incision sizes of 2.2 mm. The lens is available in 0.5 D increments from + 6.0 D to + 30.0 D and in 1.0 D increments from + 31.0 D to + 34.0 D. Lenses are available with a cylinder power of 1.0 D to 6.0 D at the IOL plane. The models SN6A-T3 (toricity 1.50 D) to SN6A-T9 (6.0 D) were implanted based on the WFK readings from the built-in iTrace toric calculator. The steep power and axis of the WFK astigmatism were calculated based on the best Zernike mathematical fit from all topological data within a 4 mm circle instead of simulating the keratometer using the iTrace topographer SimK, which uses only 4 points based on topography data on a 3 mm ring.

A clear corneal incision was made at 120° with an estimated surgically induced astigmatism (SIA) of 0.25 D created with a 2.2 mm keratome on all patients. Spherical power was calculated using biometry measurements obtained with the IOL Master 500 and calculated using the SRK/T formula. The goal in all patients was emmetropia.

### Slit-lamp marking

Before surgery, with the patient seated upright, the same experienced surgeon (SZ) marked the corneal epithelium inside the limbus of the operative eye with reference markings (e.g., 0°, 90°, and 180°) using a 26-gauge needle. Intraoperatively, a Mendez ring was used to localize the incision site and IOL placement axis. A long fine scratch was left on the corneal epithelium by a 26-gauge needle with sterile blue ink on the tip to mark the actual IOL placement axis.

### Surgical technique

Preoperatively, patients were prescribed 0.1% pranoprofen (0.1% Niflan) and 0.5% levofloxacin eyedrops for the operative eye 4 times daily for 48 h. The same experienced surgeon (SZ) performed all surgeries. A 2.2 mm primary 2-plane cataract incision and a 1.0 mm single-plane paracentesis were created. A continuous curvilinear capsulorhexis measuring approximately 5.5 mm in diameter was created. Phacoemulsification was performed using the Infiniti Vision System (Alcon Laboratories, Inc.). The folded IOLs were implanted into the capsular bag then aligned with the pre-marked axis.

### Postoperative assessment

Postoperative examinations were performed at 1 week, 1 month, and 3 months and included uncorrected and corrected distance visual acuity, intraocular pressure, subjective and objective (autorefractometry) refractions, slit-lamp evaluation, and corneal topography (Pentacam HR and iTrace). The IOL axis was assessed with toriCAM (Graham Barrett, AppStore, USA) at the slit-lamp following mydriasis.

### Astigmatism vector analysis

Postoperative refractive cylinder (adjusted to the corneal plane) and preoperative corneal WFK astigmatism were assessed by vector analysis using the Alpins method (Assort software, Assort Pty Ltd.) [1, 2]. The four main outcomes for Alpins analyses were target-induced astigmatism (TIA), surgically induced astigmatism (SIA), difference vector (DV), and correction indices (CI).

TIA was the intended magnitude and axis of astigmatic correction, where the magnitude was equivalent to preoperative corneal WFK astigmatism. SIA was defined as the actual magnitude and axis of astigmatism created during surgery. DV was the postoperative refractive cylinder (adjusted to the corneal plane). CI was defined as SIA/TIA, where values > 1 or < 1 represented overcorrection or undercorrection, respectively. The magnitude of error was the arithmetic difference between the SIA and TIA magnitudes. The magnitude of error was a positive value in overcorrection and a negative value in undercorrection. The angle of error was the axis angle difference between the SIA and TIA; it was positive or negative depending on whether the achieved correction was counterclockwise or clockwise to the intended axis, respectively. The amount of corneal incision SIA was calculated using vector analysis based on the preoperative and postoperative iTrace topography simulated keratometry data.

### Statistical analysis

All data were collected in an Excel database (version 2019, Microsoft, Redmond, WA); statistical analyses were performed with SPSS for Windows (version 23, IBM, Armonk, NY, USA). Data normality was assessed via the Kolmogorov–Smirnov test. Descriptive statistics are presented as the mean ± standard deviation or as the median (range). The iTrace WFK astigmatism results were compared with other data measured by various devices using the Student’s paired t-test. IOL rotation results were analyzed by the multiple comparison test, that is, one-way analysis of variance (ANOVA). The Bonferroni correction was applied for multiple comparisons. Differences were considered statistically significant according to the Bonferroni-corrected significance level for each comparison. A *p*-value < 0.05 was considered significant; all statistical tests were 2-sided.

## Results

Eighty-five eyes of 63 patients with cataracts and a preoperative astigmatism of 0.83–4.92 D, as assessed by iTrace, were included. Demographic data, implanted IOLs, and their power sphere are displayed in Table 1. The AcrySof Toric IOL models are displayed in Table 2.

**Table 1:** Preoperative demographics of patients studied

Characteristic	Mean ± standard deviation (range)
Age (y)	69.93 ± 13.80 (19,89)
iTrace WFK astigmatism (D)	1.91 ± 0.69 (0.83,4.92)
IOL power sphere (D)	19.83 ± 3.38 (9, 29)
Predicted spherical equivalent (D)	−0.26 ± 0.18 (− 0.77, 0.12)
Axial length (mm)	23.68 ± 1.23 (21.44, 28.38)
Anterior chamber depth (mm)	3.04 ± 0.41 (2.31, 4.10)

*D* diopter, *WFK* wavefront keratometric, *IOL* intraocular lens

**Table 2:** Percentage of the toric IOL model

Model	Number	%
SN6AT3	14	16.5
SN6AT4	34	40.0
SN6AT5	24	28.2
SN6AT6	9	10.6
SN6AT7	2	2.4
SN6AT8	1	1.2
SN6AT9	1	1.2
*Total*	*85*	*100.0*

### Visual acuity and refraction

**Fig. 1:**
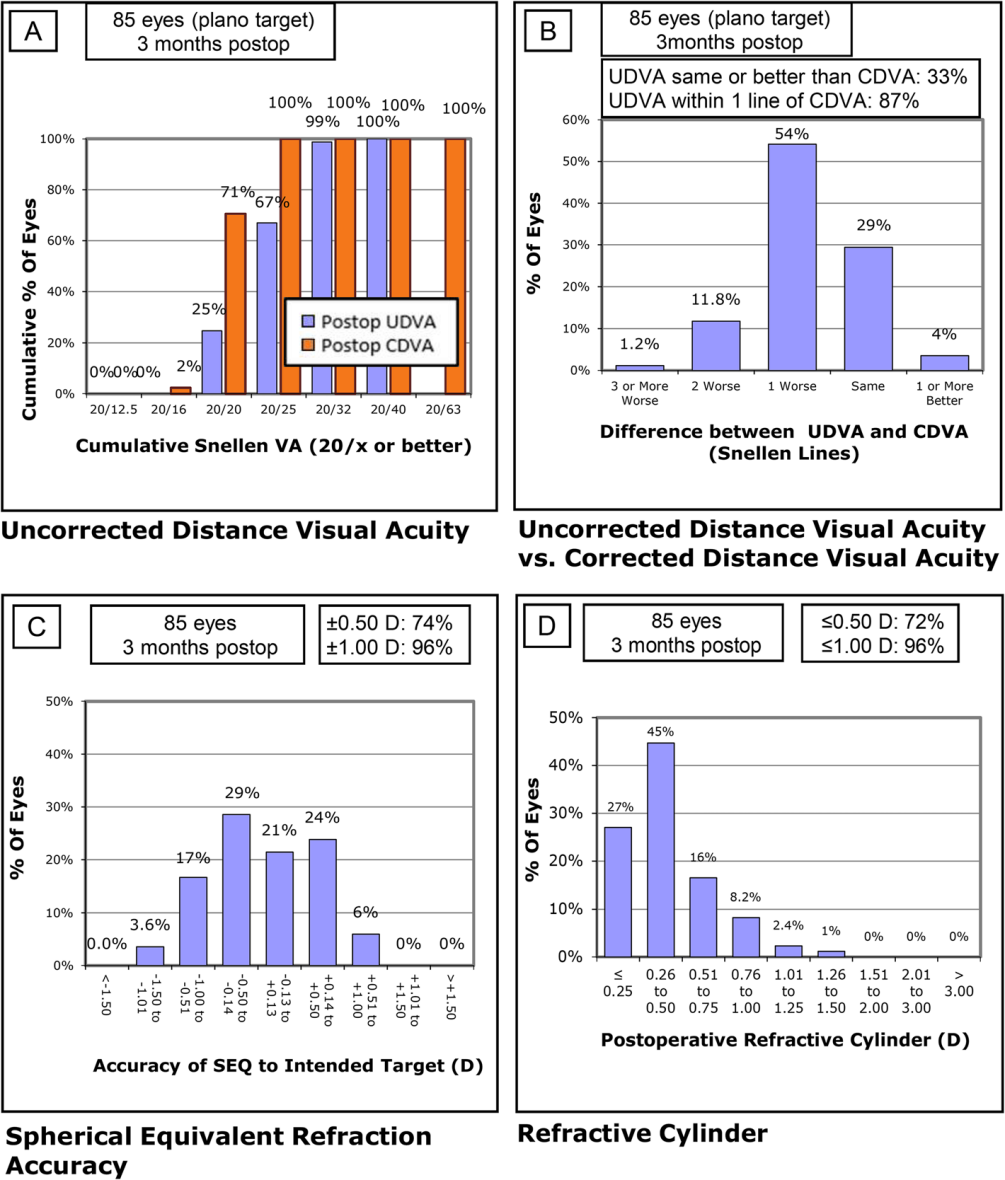
Refractive and visual outcomes. CDVA, corrected distance visual acuity; UDVA, uncorrected distance visual acuity; VA, visual acuity; postop, postoperative; preop, preoperative; SEQ, spherical equivalent

Three-month postoperative uncorrected and corrected distance visual acuity data are shown in Fig. 1a while a histogram of differences between these measures is displayed in Fig. 1b; these data reveal the surgery efficacy. A histogram comparing the postoperative spherical equivalent refraction to the intended target is displayed in Fig. 1c, revealing the surgery predictability. The postoperative refractive cylinder is displayed in Fig. 1d and Table 3; notably, 71.8% were ≤ 0.50 D and 88.2% were ≤ 0.75 D.

**Table 3:** Cumulative magnitudes of the preoperative corneal astigmatism and postoperative refractive astigmatism

Diopter	Preoperative corneal		Postoperative refractive	
	Number	%	Number	%
≤ 0.25	0	0	23	27.1
≤ 0.50	0	0	61	71.8
≤ 0.75	0	0	75	88.2
≤ 1.00	3	3.5	82	96.5
≤ 1.25	14	16.5	84	98.8
≤ 1.50	25	29.4	85	100
≤ 2.00	52	61.2	85	100
≤ 3.00	81	95.3	85	100
≤ 5.00	85	100	85	100

### Vector analysis

**Fig. 2:**
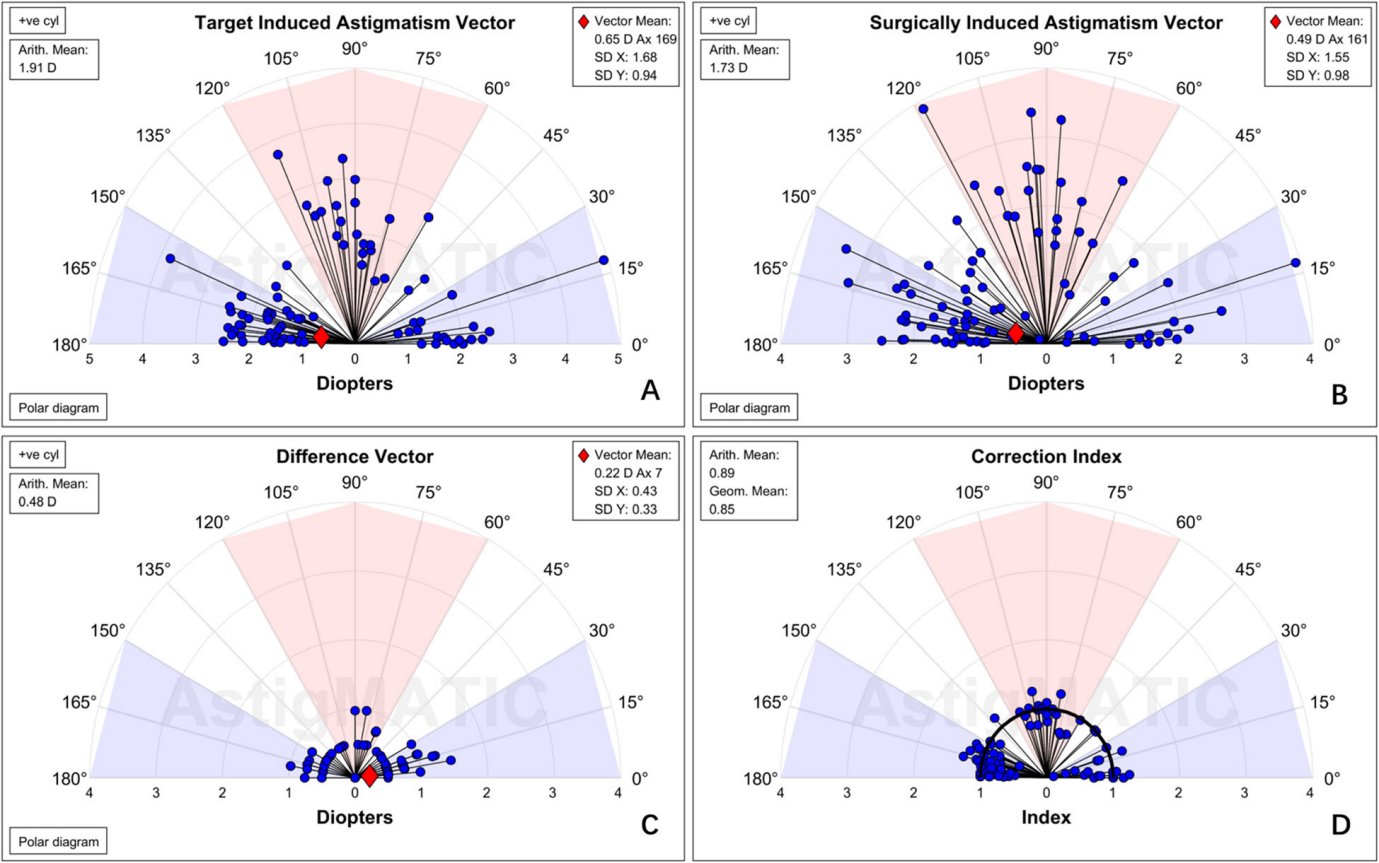
Single-angle polar plots for the **a** target induced astigmatism vector, **b** surgically induced astigmatism vector, **c** difference vector, and **d** correction index are shown. The vector means are plotted as a red cross (calculated in double-angle vector space) and the standard deviations (SDs) for the X and Y axes are displayed in the call-out box. D, diopters

Vector analysis using the Alpins method was performed at the 3-month follow-up examination (Table 4, Fig. 2). The average arithmetic for SIA was 1.73 ± 0.77 D (0.13, 3.92) and the centroid was 0.49@161° ± 1.83D (Fig. 2, Table 4). The average arithmetic remaining astigmatism was 0.48 ± 0.34 D (0.00, 1.46), and the centroid was 0.22@7° ± 0.55 D. The average of CI was 0.89 ± 0.22, revealing a minimal undercorrection (Fig. 2, Table 4).

**Table 4:** Vector data for the toric intraocular lens

Parameter, 3 months postoperative	Mean absolute	Centroid
Target induced astigmatism (D)	1.91 ± 0.69 (0.83, 4.92)	0.65@169° ± 1.93D
Surgically induced astigmatism (D)	1.73 ± 0.77 (0.13, 3.92)	0.49@161° ± 1.83D
Difference vector (D)	0.48 ± 0.34 (0.00, 1.46)	0.22@7° ± 0.55D
Magnitude of error (D)	0.18 ± 0.35 (− 0.70, 1.07)	
Angle of error (°)	−5.89 ± 10.67 (− 44.12, 10.06)	
Correction index	0.89 ± 0.22 (0.1, 1.29)	

Data are presented as the mean ± standard deviation (range)

*D* diopter

**Fig. 3:**
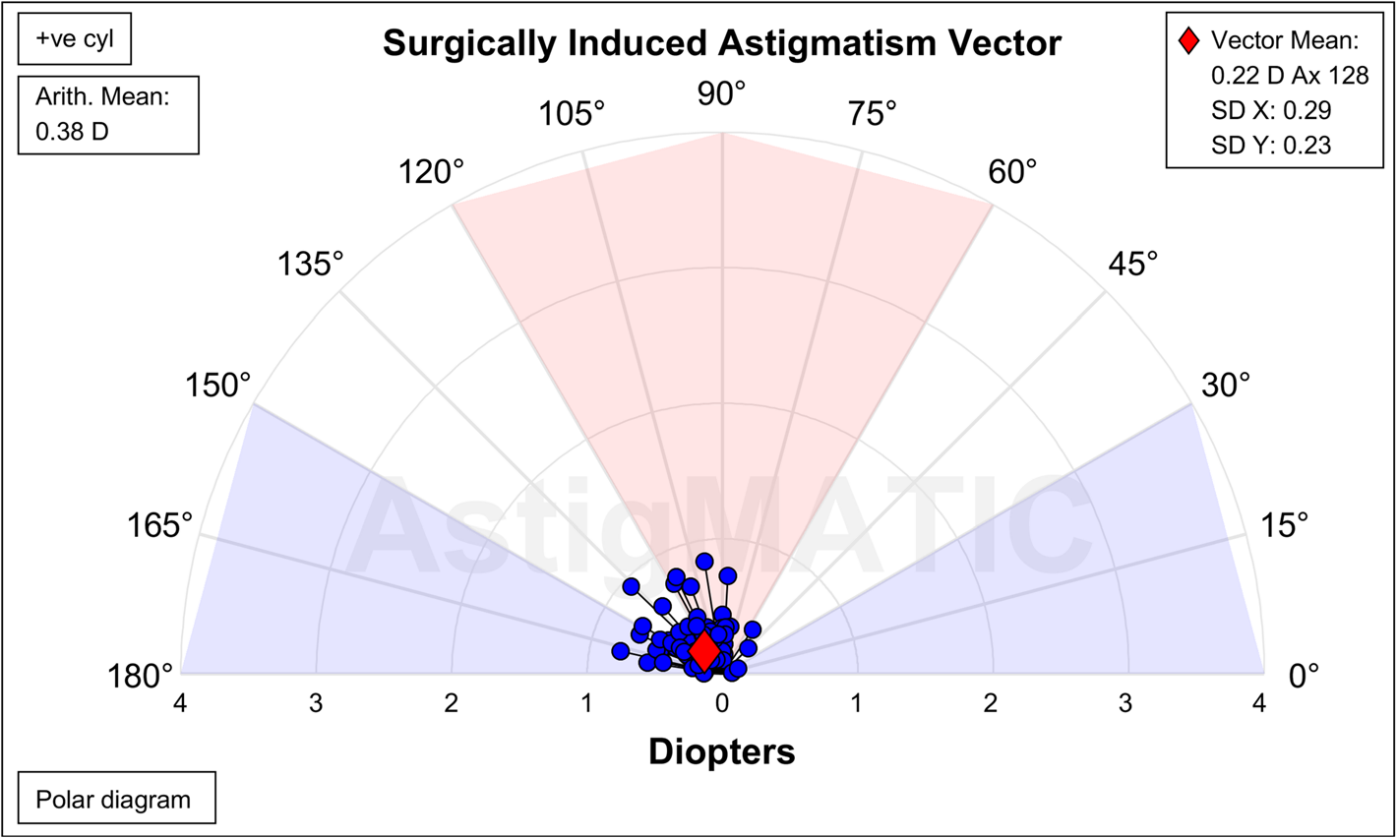
Vectors of SIA caused by corneal incisions. SIA, surgically induced astigmatism

All patients received a clear corneal incision of 2.2 mm at 120°. Comparing pre- and postoperative K values (measured by SimK), the average arithmetic surgically induced corneal astigmatism was 0.38D ± 0.20D (0.07, 0.94), and the centroid was 0.22@128° ± 0.37 D (Fig. 3).

### Comparison of corneal astigmatism measured with different devices

iTrace WFK, IOL Master SimK, and Pentacam WFK showed a high mean astigmatism. Compared to the iTrace WFK, IOL Master SimK, and Pentacam WFK had no significant statistical differences (*p* = 0.456 and *p* = 0.510, respectively) (Table 5).

**Table 5:** Comparison of corneal astigmatism measured by iTrace wavefront aberrometry and other techniques

Corneal astigmatism (D)	Mean ± standard deviation (range)	P
iTrace WFK	1.91 ± 0.69 (0.83, 4.92)	
iTrace SimK	1.77 ± 0.68 (0.51, 4.59)	0.000
IOL Master SimK	1.88 ± 0.74 (0.59, 4.34)	0.456
Pentacam SimK	1.69 ± 0.75 (0.3, 4.1)	0.000
Pentacam WFK	1.89 ± 0.72 (0.40, 4.70)	0.510
Pentacam AK 3 mm Zone	1.65 ± 0.74 (0.50, 4.40)	0.000
Pentacam AK 4 mm Zone	1.65 ± 0.70 (0.60, 4.40)	0.000
Pentacam TRP	1.74 ± 0.73 (0.2, 4.4)	0.000

A Student’s paired t-test was used for statistical analysis

*WFK* wavefront keratometry, *SimK* simulated keratometry, *AK* axial keratometry, *TRP* total refractive power, *D* diopter

### Intraocular lens rotation

**Fig. 4:**
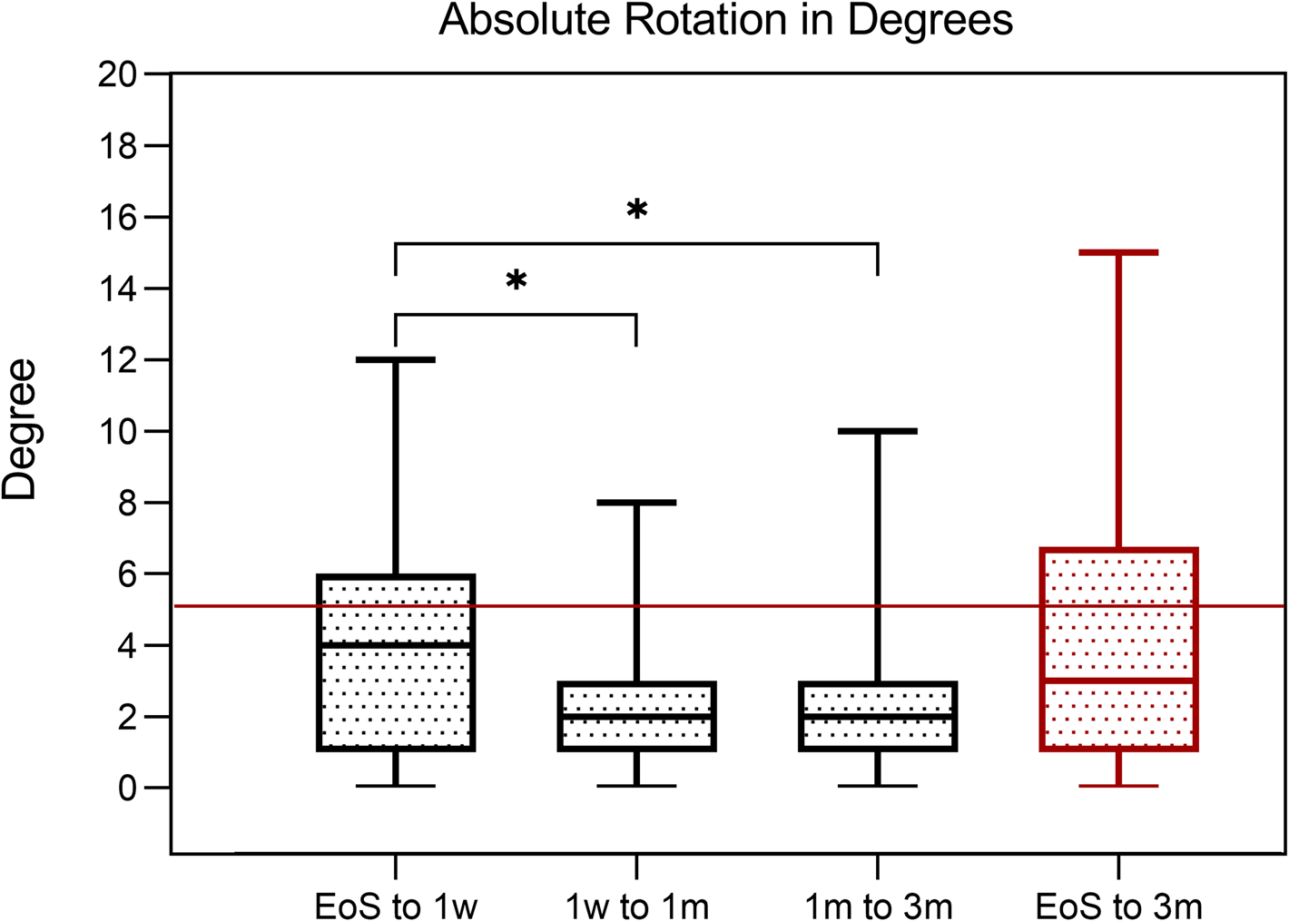
Absolute rotation in degrees from time-point to time-point. Within the first week, rotation was statistically significantly increased compared with all other time-points (*p* = 0.003; *p* = 0.002, one-way ANOVA). EOS, end of surgery; 1w, 1 week; 1 m, 1 month; 3 m, 3 months

All patients underwent mydriasis during follow-up and the IOL axial position was measured. Table 6 shows detailed rotation data through all time-steps. IOL rotation within the first week after surgery was significantly higher compared with all other time-points (Fig. 4) (*p* = 0.003 and *p* = 0.002, respectively). After 3 months, 58 (68.2%) IOLs showed < 5.0° rotation.

**Table 6:** Rotation of toric intraocular lens

	Absolute intraocular lens rotation			
	Median (range) (°)	Mean ± standard deviation (°)	≤ 5°(%)	≤ 10°(%)
1 week to the end of surgery	4 (0–12)	4.13 ± 3.27	68.3	95
1 month to 1 week	2 (0–8)	2.16 ± 1.79	94.1	100
3 months to 1 month	2 (0–10)	2.10 ± 1.96	94.2	100
3 months to the end of surgery	3 (0–15)	3.94 ± 3.85	68.2	93.8

Kruskal-Wallis test and Dunn’s multiple comparisons test was used for statistical analysis

## Discussion

Factors influencing residual refractive astigmatism after cataract surgery with toric IOLs include accurate preoperative corneal astigmatism measurements, variability in the magnitude and direction of corneal incision SIA, the effects of different toric calculators, the rotational stability of different toric IOLs [8], and reported lens tilt [9]. Several diagnostic devices based on different technologies are available to measure preoperative corneal power and astigmatism, including manual and automated keratometers; Placido-based corneal, point-source color light emitting diode, Scheimpflug image-based, and scanning-slit corneal topographers; low-coherence reflectometers; and intraoperative aberrometers [10–13]. However, none of these methods are currently considered the gold standard. In the present study, the outcomes of toric IOL implantation based on iTrace WFK and its built-in toric calculator were investigated. The iTrace WFK astigmatism is anecdotally described as being more accurate than simulated keratometry, but no prior research was clearly available to document this observation.

In this study, preoperative mean corneal topographic astigmatism was 1.91 diopters (D) ± 0.69. Postoperative mean refractive astigmatism decreased significantly to 0.48 D ± 0.34. 88.2% of the postoperative residual astigmatism were ≤ 0.75 D and 71.8% ≤ 0.5 D. These outcomes are very similar to previous studies. Potvin et al. evaluated clinical outcomes of patients whose toric IOL calculations were based on the Lenstar LS900 dual zone automated keratometer and found that 76% of eyes had ≤0.50 D of astigmatism after 3 months [14]. Results using the Barrett toric calculator show 72–80% of the cases with a residual refractive astigmatism of 0.5 D or less [15, 16].

The magnitude and direction of corneal incision SIA are essential in surgical planning. The average arithmetic corneal SIA in this study of 0.38 D ± 0.20 D (0.07, 0.94) and the centroid of 0.22@128° ± 0.37 D were close to the predicted value 0.25 D.

Among the mean corneal astigmatism measured with 3 devices (iTrace, IOL Master, and Pentacam) a higher mean was measured with iTrace WFK than with the axial keratometry of the 3-mm (4-mm) corneal zone, the simulated keratometric astigmatism and total refractive power measured with Pentacam; however, the mean was similar to the IOL Master simulated keratometric astigmatism and WFK within a 4-mm zone calculated with Pentacam. Park et al. showed the IOL Master corneal astigmatism measurements were higher than those calculated by iTrace wavefront aberrometry and simulated astigmatism [17]. The present findings confirm that IOL Master has a tendency to provide a higher value and show a similar trend of iTrace WFK and Pentacam WFK astigmatism.

Optimal astigmatism correction with a toric IOL requires both accurate surgical alignment and rotation stability. Several factors can influence postoperative rotational stability and IOL misalignment, such as the design and material of toric IOLs, the ophthalmic visco surgical device inside the capsular bag after surgery, large white-to-white measurements, and inaccurate preoperative axis marking [18]. A needle was used to mark the axis of toric IOL in the present study to decrease variation due to broad marking and reduce the possibility of spreading and washing out the dye due to tear flow. The method is similar with that proposed by Bhandari and Nath [19]. Their study found the postoperative mean IOL deviation at 1 day and 1 month was 5.7 ± 6.5° and 4.7 ± 5.6°, respectively. Furthermore, the postoperative median IOL misalignment was 3° at 1 day and 1 month [19], consistent with the present outcomes. A recent study shows that 28% of the mean toric IOL axis misalignment measured postoperatively is caused by intraoperative misalignment rather than postoperative rotation [20]; also, rotation between 1 h and 1 day postoperatively was rare [20]. At 1 postoperative year, the mean toric IOL axis misalignment was 6.67°, of which 1.87° were caused by surgical misalignment and 4.80° were caused by toric IOL rotation.^19^ Previous research suggested that a digital overlay system, including intraoperative wavefront aberrometry, and a digital marking system results in lower intraoperative misalignment and postoperative astigmatism than traditional manual marking [18, 21, 22]. Mayer et al. found statistically significant differences between manual marking and digital marking with better toric IOL alignment in the digital marking group (2.0 degrees versus 3.4 degrees) [18]. In this study, the intended axis was used as the baseline instead of the actual axis at which the IOL was positioned. Digital navigation was not used to the mark toric IOL axis. The average 1-week postoperative rotation median of the toric IOL axis was 4 degrees; therefore, part of the toric IOL misalignment might be caused by interoperative misalignment.

Pallas et al. found that the toriCAM application, used in the present study, potentially can significantly reduce reference marking errors, thus potentially improving the accuracy of both marking methods; hence, toriCAM use appears to be of greater benefit to the freehand than the slit-lamp method of marking [23].

There are several limitations to this study. Twenty-two patients had both eyes enrolled in this study, which represents a study limitation. A needle was used to mark the toric IOL axis without digital marking system assistance. Also, the toric IOL axis was not able to be observed accurately at the end of the surgery. Therefore, it was not possible to distinguish between intraoperative toric IOL axis misalignment and postoperative rotation. we suggest that further studies should be carried out to compare outcomes or theoretical outcomes between the iTrace WF keratometric astigmatism and the other measurement methods. The study showed that iTrace built-in toric calculator with wavefront keratometric astigmatism was safe and effective for toric IOL planning, but no comparison between clinical or theoretical outcomes between the iTrace WF keratometric astigmatism and the other measurement methods, further studies should be done to find out which method is better.

## Conclusions

The iTrace wavefront aberrometry of cornea calculated steep power and axis based on the best Zernike mathematical fit from all topo data within 4 mm circle. It included more information of the central corneal topography data than simK. To the best of our knowledge, this is the first study evaluating the clinical outcomes of using iTrace wavefront keratometric readings to plan a toric IOL implantation.

The results indicated that toric IOL planning according to iTrace WFK values and the built-in calculator received comparable clinical outcomes to the previous studies. It proved the efficacy and safety of toric IOL planning based on iTrace.

## Data Availability

The datasets used and/or analyzed during the current study available from the corresponding author on reasonable request.

## Abbreviations

IOL: Intraocular lens
WFK: Wavefront keratometry
SimK: Simulated keratometry
AK: Axial keratometry
TRP: Total refractive power
SIA: Surgically induced astigmatism
TIA: Target-induced astigmatism
DV: Difference vector
CI: Correction index
CDVA: Corrected distance visual acuity
UDVA: Uncorrected distance visual acuity
VA: Visual acuity
postop: Postoperative
preop: Preoperative
SEQ: Spherical equivalent
D: Diopter
EOS: End of surgery
1w: 1 week
1 m: 1 month
3 m: 3 months
